# High Sensitive pH Sensor Based on AlInN/GaN Heterostructure Transistor

**DOI:** 10.3390/s18051314

**Published:** 2018-04-24

**Authors:** Yan Dong, Dong-Hyeok Son, Quan Dai, Jun-Hyeok Lee, Chul-Ho Won, Jeong-Gil Kim, Dunjun Chen, Jung-Hee Lee, Hai Lu, Rong Zhang, Youdou Zheng

**Affiliations:** 1 School of Electronic Science and Engineering, Nanjing University, Nanjing 210023, China; yandong199@smail.nju.edu.cn (Y.D.); hailu@nju.edu.cn (H.L.); rzhang@nju.edu.cn (R.Z.); ydzheng@nju.edu.cn (Y.Z.); 2 School of Electronics Engineering, Kyungpook National University, Daegu 702-701, Korea; dhson@ee.knu.ac.kr (D.-H.S.); dqfight@hotmail.com (Q.D.); ljh0621@knu.ac.kr (J.-H.L.); chwon@ee.knu.ac.kr (C.-H.W.); jgkims2@ee.knu.ac.kr (J.-G.K)

**Keywords:** AlInN/GaN, AlGaN, HEMT, pH sensor, open gate geometry

## Abstract

The AlInN/GaN high-electron-mobility-transistor (HEMT) indicates better performances compared with the traditional AlGaN/GaN HEMTs. The present work investigated the pH sensor functionality of an analogous HEMT AlInN/GaN device with an open gate. It was shown that the Al_0.83_In_0.17_N/GaN device demonstrates excellent pH sense functionality in aqueous solutions, exhibiting higher sensitivity (−30.83 μA/pH for AlInN/GaN and −4.6 μA/pH for AlGaN/GaN) and a faster response time, lower degradation and good stability with respect to the AlGaN/GaN device, which is attributed to higher two-dimensional electron gas (2DEG) density and a thinner barrier layer in Al_0.83_In_0.17_N/GaN owning to lattice matching. On the other hand, the open gate geometry was found to affect the pH sensitivity obviously. Properly increasing the width and shortening the length of the open gate area could enhance the sensitivity. However, when the open gate width is too larger or too small, the pH sensitivity would be suppressed conversely. Designing an optimal ratio of the width to the length is important for achieving high sensitivity. This work suggests that the AlInN/GaN-based 2DEG carrier modulated devices would be good candidates for high-performance pH sensors and other related applications.

## 1. Introduction

GaN-based devices have attracted much attention for many different applications including high-frequency/power devices, super lattice high-speed switch devices, light-emitting diodes, photoelectric detectors, various sensors, etc. The GaN-based heterostructure hosts a two-dimensional gas (2DEG) channel with high sheet carrier concentration and high electron mobility, a wide band gap, high carrier velocity saturation and good chemical stability [1,2,3,4,5]. On the other hand, gallium nitride material devices can be operated at high temperatures up to 500 °C owing to its wide band gap (3.4 eV for GaN versus 1.1 eV for Si) [6], enabling good performance in spacecraft, satellite radar or other electronic equipment working in high temperature environments without using additional cooling segment, therefore simplifying the complexity of the integrated circuit, reducing spacecraft launch weights and increasing satellite functional capabilities. In addition, strong chemical stability and good biological compatibility make GaN-based devices as good candidates for fabricating ion sensors. Solutions’ pH is essential in all biological and most chemical reactions. How to probe pH sensitively has been demonstrated to be of particular paramount importance in a large number of fields, for example environmental science, healthcare, as well as acidosis. Compared with other sensors based on conventional semiconductor materials, all the layers in the GaN-based devices are undoped [7]. The two-dimensional gas (2DEG) channel of GaN-based devices is induced by spontaneous and piezoelectric polarization, which can be balanced by positive charges or negative charges on the surface, so the 2DEG density of GaN-based devices is extremely sensitive to its surroundings (gas, chemical ion, pressure and biomolecule) [7]. Besides, the additional sensitive membrane on the surface is not necessary for GaN-based ion sensors [8,9], which can save the process time and simplify processes’ complexity, therefore reducing the product cost. The sensitivity to the surface charge results in the a good application of these ion sensors as pH value monitors, which has been widely adopted by using the traditional AlGaN/GaN HEMTs [9,10,11].

Compared with AlGaN, AlInN is in principle a better candidate material for these surface charge sensitive sensors because a much smaller thickness of the barrier layer is required to achieve the same 2DEG density as that in AlGaN/GaN HEMTs, due to stronger spontaneous polarization and larger conduction band discontinuity at the AlInN/GaN interface [12]. Besides, better interface quality with much lower dislocation density can be realized with reduced interfacial stress when the In composition is at 17.3% (lattice matching with GaN) [13]. According to these advantages, the excellent performances of devices of the AlInN/GaN heterostructure have been reported over the past few decades [14,15,16,17,18]. However, only a few works on ion sensors are based on the AlInN/GaN heterostructure [19,20,21,22]. T. Brazzini firstly investigated the performance of a GaN-caped AlInN/AlN/GaN field effect transistor as the pH-sensing application and demonstrated a drain current pH sensitivity from −1.37 μA/pH to −4.16 μA/pH, dependent on the device geometry. This displayed the potential of pH sensor application of AlInN in spite of no comparison experiments with the traditional AlGaN/GaN devices having been done and only three pH values of 4, 7 and 10 having been evaluated, and the GaN cap layer also may have a contribution to the sensitivity [19]. In other existing related investigations, AlInN/GaN HEMTs have been used to try to detect deoxyribonucleic acid (DNA) hybridization, demonstrating higher sensitivity in comparison with the AlGaN/GaN HEMT biosensor [19]. Alternately, the AlInN/GaN sensor similarly exhibits better sensitivity than that of the AlGaN/GaN sensor in phosphate detection. The response was ultrasensitive with a limit below 0.02 mg/L, as well as the specific recognition of the phosphate anion [20]. The polar liquid and NH_3_ gas sensitivity of the AlInN/GaN interface also were reported [21,22]. In the present work, we systematically investigated the pH value-sensitive performances of AlInN/GaN heterostructure devices. High sensitivity with linear dependence on the pH value was achieved up to 30.83 μA/pH, seven-times that of the AlGaN/GaN device; for comparison, also much larger than that reported by T. Brzzini. The sensors demonstrated high stability. The effect of open gate geometry on the pH value sensitivity and the sensing mechanism also were unveiled and discussed.

## 2. Experimental and Discussion

**Figure 1:**
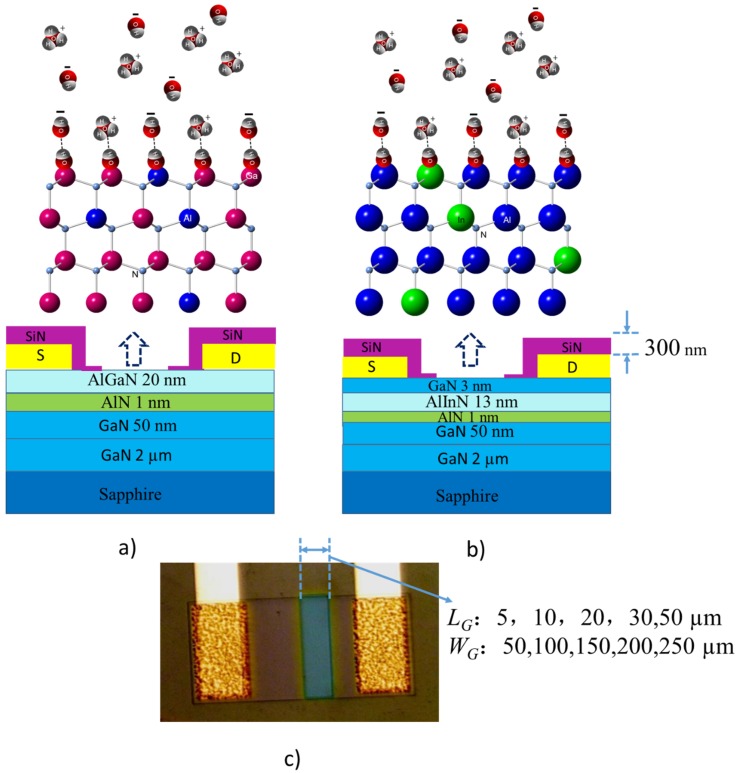
(**a**) Schematic illustration of the AlGaN/GaN heterostructure open gate pH sensor and the graphic representation of reactions between the POH groups on the sensing surface and other ions in the aqueous solution, where Ga, Al and N atoms are represented by pink, blue and light blue. (**b**) Schematic illustration of the AlInN/GaN heterostructure open gate pH sensor and the graphic representation of the reactions between the POH groups on the sensing surface and other ions in the aqueous solution, where Al, In and N atoms are represented by blue, green and light blue. The hydrone (water molecules), hydrion (H ion) and hydroxyl ion are shown; O and H are represented by red and gray. (**c**) The top view of one fabricated device.

The analogous HEMT structure with an open gate consists of an Metal-organic Chemical Vapor Deposition (MOCVD)-grown AlGaN/GaN layer or AlInN/GaN layer, and they were grown on a sapphire substrate (shown in Figure 1a,b). The undoped GaN buffer layer (high-Resistance and 2 μm) was grown on the sapphire firstly, which is basically necessary to achieve a uniform Ga termination polarity fir the GaN epitaxial-layer and improve the crystal quality of the following GaN channel layer with a 50-nm thickness; therefore, improving the density of 2DEG. A 1 nm-thick AlN spacer layer also was adopted to improve the device quality. The AlGaN layer thickness is 20 nm, with an Al composition 0.25, for the AlGaN/GaN devices, and the AlInN layer is 13 nm thick with an in composition of 0.17. They are the barrier layers and were capped by a GaN layer with a thickness of 3 nm to realize a more uniform surface of higher quality of AlInN/GaN. It is worth mentioning that the GaN cap layer will be removed in a later process in order to evaluate the sensing ability of the AlInN surface.

The standard four probes’ resistance and Hall effect measurement were adopted to characterize the basic electric properties of the devices. The prepared AlGaN/GaN device has sheet resistance, carrier concentrations and mobility with 364.2 Ω/□, 1.02 × 10^13^ cm^−2^ and 1680 cm^2^/V·S, respectively. The prepared AlInN/GaN device has sheet resistance, carrier concentrations and mobility of 282.5 Ω/□, 2.34 × 10^13^ cm^−2^ and 944 cm^2^/V·S, respectively. Two reasons are attributed to the lower carried mobility in the AlInN/GaN devices, in comparison with the AlGaN/GaN. First, the AlInN/GaN heterostructure has a higher 2DEG density, which will increase scattering and so degrade the carrier mobility. Second, it is much harder to obtain a good quality AlInN/GaN heterostructure due to the higher amount of aluminum in comparison with AlGaN/GaN, because AlN and InN have contradictions in growth conditions; the interface between AlInN and GaN gas greater roughness. The open gate device was fabricated by the conventional integrated circuit (IC) process. The isolation and active region were patterned by using inductively-coupled plasma (ICP) etching, and a 20-nm SiN layer was deposited as the isolated layer to protect the AlInN or AlGaN layer from heat damage during the following Ohmic contact process and high-temperature rapid thermal process (RTP). The Ohmic contact of Ti/Al/Ni/Au (25/160/40/100 nm) layers was formed using an electron-beam evaporator (E-beam). After that, the RTP process was adopted to obtain good Ohm contact. The Ti/Al/Ti (40/100/30 nm) metal layers as the pad contact were deposited also by E-beam. The final protective layer with a 300 nm-thick layer of SiN was deposited to isolate the device form acid or alkaline solutions when it works. Finally, standard buffer solution (BOE: HF+NH_4_F) was used to open the sensitive area and pad contact.

The horizontal cross-sections of the AlInN/GaN and AlGaN/GaN devices’ structures are shown in Figure 1a,b. Figure 1c shows a photograph of one device. Two kinds of HEMT structures (AlGaN/GaN, AlInN/GaN) with different ratios of *W*_G_/*L*_G_ (*W*_G_: the open gate width; *L*_G_: the open gate length) were prepared. The open gate length *L*_G_ is 5, 10, 20, 30 and 50 μm, respectively; the open gate width *W*_G_ is 50, 100, 150, 200 and 250 μm, respectively.

In order to assess the impact of device open gate size on the pH sensing ability and measure the pH value sensing performance of the devices, commercial standard MRS-41089 solutions (a mixed solution of HCl and NaOH in DI water) with different pH values was dropped on the sensing region of the devices, and then, the I–V data were probed by an Agilent B1500A (Santa Clara, CA, USA) semiconductor device analyzer. The pH sensitivity is characterized by the current variation of the device before and after immersing in the solution.

## 3. Results

**Figure 2:**
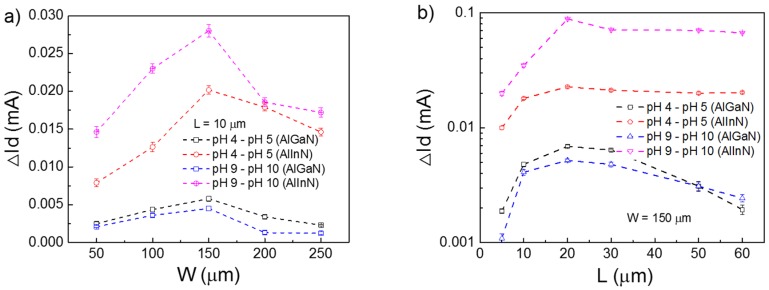
Open gate geometry dependence of the output current of the devices. (**a**) Devices with open gate length *L* of 10 μm, but with different widths; (**b**) devices with open gate width *W* of 150 μm, but with different lengths.

First, we optimized the gate geometry of the devices. As shown in Figure 2a, the current variation response on the pH value changed from both pH 4–pH 5 and pH 9–pH 10 maximum peaks at the gate width of 150 μm, for devices with fixed gate lengths of 10 μm, independent of the barrier materials. With fixed gate width of 150 μm, the devices exhibit the largest current variation when their gate lengths are 20 μm, as shown in Figure 2b. Therefore, a device with the gate geometry as 150 × 20 μm^2^ has the best response on pH value change, whether in acid or alkali solution.

**Figure 3:**
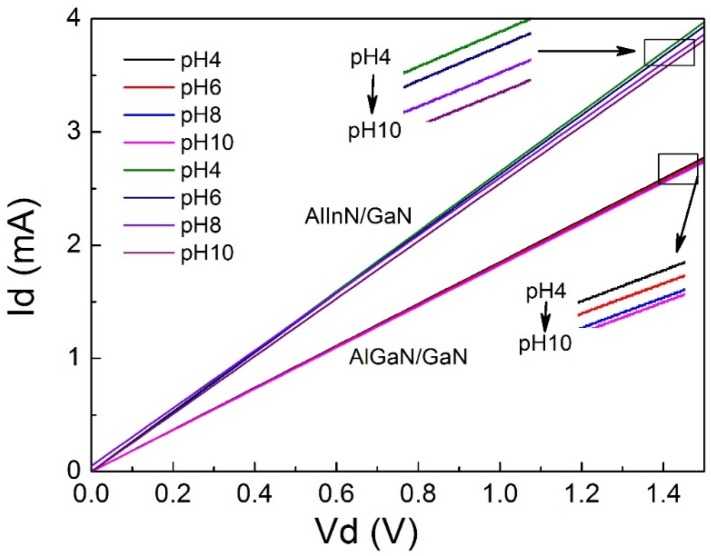
The *I_d_*-*V_d_* performance of two kinds of gateless HEMT structure sensors; they are AlInN/GaN and AlGaN/GaN heterostructures, respectively.

**Figure 4:**
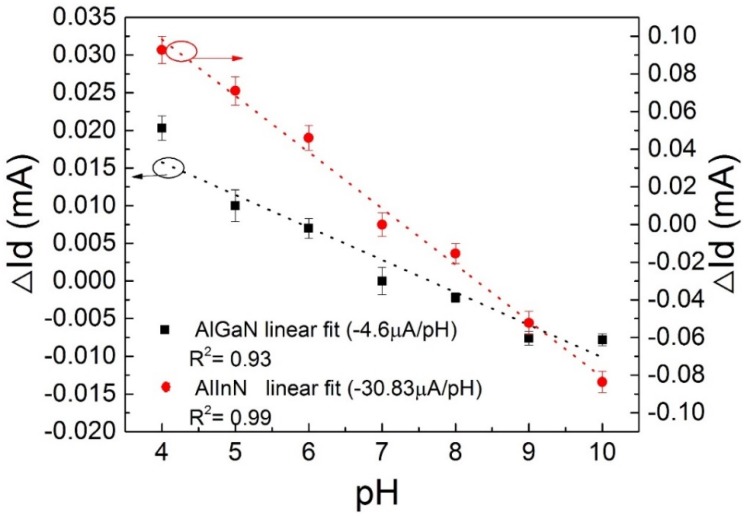
The current variation (∆*I_ds_*) as a function of pH and linear fits for the AlInN/GaN and AlGa/GaN heterostructure devices; the *V_ds_* fix to 1 V.

Based on the optimized gate geometry of 150 × 20 μm^2^, the sensitive behaviors of the devices were characterized as a function of the wide range of pH values and the wide range of driven voltages. It was shown that the devices bear a linear correlation of *I_ds_*-*V_ds_* curves in different pH aqueous solutions (from pH 4–pH 10), for both the AlGaN/GaN device and the AlInN/GaN device. Compared with the AlGaN/GaN device, the AlInN/GaN device shows higher current, in agreement with its higher density of 2DEG. Notably, *I_ds_* decreases with the pH value increasing, and this can be seen from Figure 3, as well. The current variation of the devices was extracted from Figure 3, at the driven voltage *V_ds_* = 1 V, and depicted in Figure 4. The current variation is a linear function of pH value, with a negative slope, which gives the pH value sensitivity of the device. They are −4.6 μA/pH for the AlGaN/GaN device and −30.83 μA/pH for the AlInN/GaN device, indicating a big improvement of the sensitivity for the AlInN/GaN case. Compared with other pH value sensing devices, the sensitivity of the AlInN/GaN device −30.83 μA/pH is absolutely much larger. For example, the sensitivity of a PbO thin film pH sensor is reported at 1.16 μA/pH [21] and 1.85 μA/pH for a V_2_O_5_/WO_3_ thin film pH sensor [22]. The probed current fluctuation of the present AlInN/GaN sensor is at a scale of 0.1 μA, and the precise of the pH value can be distinguished; therefore, it can be roughly deduced as pH 0.003 according to the sensitivity, which is much larger than the best precise pH 0.01 of the available commercial pH meter.

**Figure 5:**
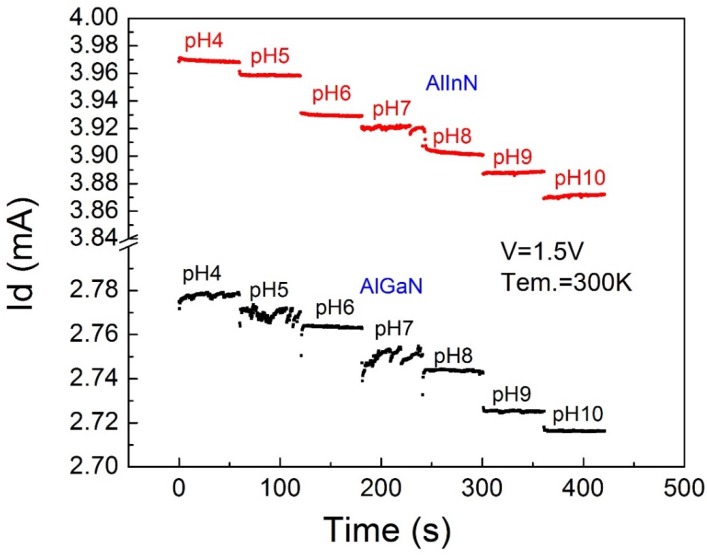
The drain to source current *I_ds_* of two structures’ sensors during real-time measurement of the pH value change from 4–10 when the *V_ds_* equal to 1.5 V with a time interval of 1 s. The numbers indicate the corresponding pH values in a mixed solution of HCl and NaOH in water and the pH values measured with a calibrated pH meter.

**Figure 6:**
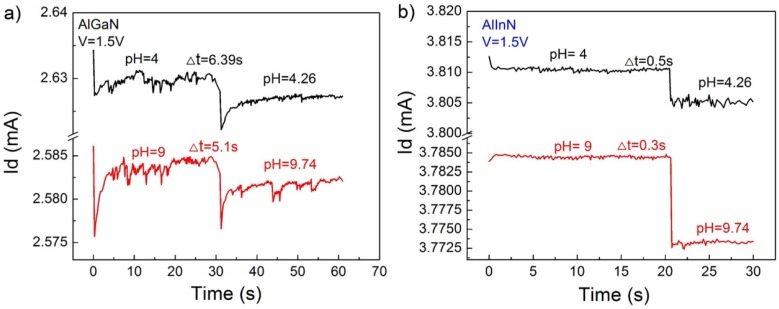
The transient response time of two structures’ devices when the solution pH value changes from 4–4.26 and from 9–9.74. (**a**) AlGaN/GaN heterostructure device; (**b**) AlInN/GaN heterostructure device.

We further characterized the time response of the pH value of the devices; the results are demonstrated in Figure 5 and Figure 6. Figure 5 evaluates the short time stability of the devices; the current output is basically decreased with the increase of pH value. The AlInN device showed better performance than the AlGaN device, as mentioned in Figure 3. Roughly, the current response is stable during a time period of 50 s after loading the solutions, which reinforces the practical applicability of the devices. Carefully checking the transient response defined as the behavior in a very short duration after solution dropping, the current response of the AlInN/GaN device is gradually decreased and then goes into a stable state. For the AlGaN/GaN device, it was increased at a low pH value, but decreased at a high pH value. We do not argue this would be an intrinsic feature of the device, which perhaps was influenced by the experimental details. However, one thing that can be concluded is that the transient response time for the AlInN case is much smaller than that of the AlGaN device. As shown in Figure 6, for the AlInN/GaN structure device, the Δ*t* is 0.5 s and 0.3 s respectively, when the solution pH value was changed from 4–4.26 or from 9–9.74; however, for the AlGaN/GaN structure device, the transient response time is 6.39 s when the solution pH value was changed from 4–4.26 and 5.1 s when the solution pH value was changed from 9–9.74, respectively. The response speed of the AlInN/GaN sensor is about 100 times that for a commercial pH meter shown in [23]. This faster response behavior of the AlInN device is attributed to its thinner barrier layer, endowing the sensor with higher efficiency and lower power dissipation.

**Figure 7:**
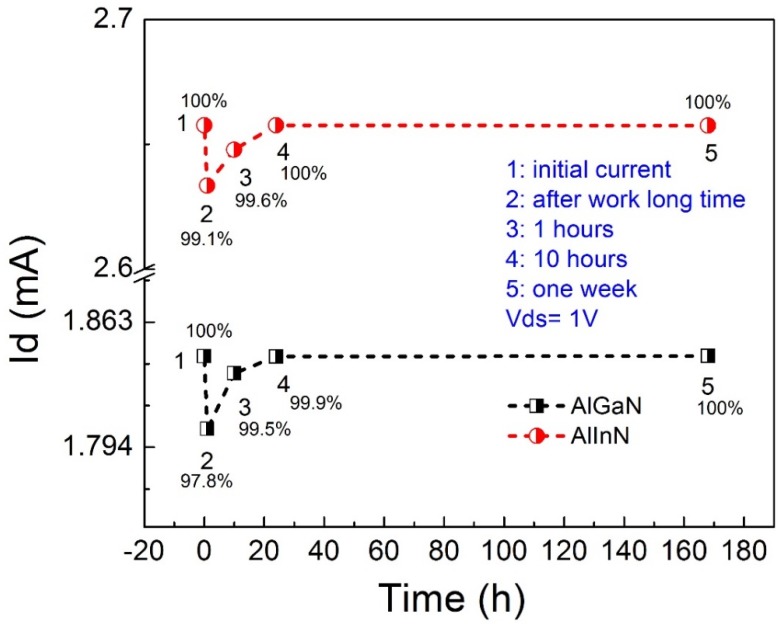
For AlGaN/GaN and AlInN/GaN structure devices, their performance degradation and the recovery condition after working in a solution for a long time.

As for the pH sensor, it must possess good performance stability after measurements, which means the working solution cannot introduce permanent damage to the device. To evaluate the effect of the working solution on the device performance, we measured the current of the devices for different procedures, as shown in Figure 7. The initial current at Point 1 represents the current of the device without the contacting solution. After a long time working with immersion in solution (immersion time approximately equal to 8 h), the device was washed and dried by air flow, then the current was measured, corresponding to Point 2. Obviously, the device showed degradation due to the effect of the solution in the working procedure. For the AlGaN/GaN device, the current was down to 97.8% of the initial current. It was 99.1% for the AlInN/GaN device. Fortunately, this degradation is not permanent. The current of the device without contacting solution was slowly recovered. It has recovered to 99.5% for the AlGaN case and 99.6% for the AlInN case, after one hour. After 10 h, the current of the devices has been nearly fully recovered. After one week, the performance still remained. Therefore, we can draw the conclusion that the devices have good chemical resistance to the solutions we used in the experiments. The AlInN/GaN device demonstrated better stability than the AlGaN/GaN device.

## 4. Discussion

We have displayed that the devices herein studied can distinguish different pH value levels with a high linear sensitivity. However, as deficiency remains in precisely understanding the latent mechanism of the response so far from the viewpoint, whether for chemistry or physics, although some work has been done [24,25,26]. The site-binding model is often adopted to roughly interpret the pH-sensing process as an absorption effect of protons (H^+^) or hydroxyl (OH^−^) ions by the hydroxyl groups on the device sensing surface, which will form positive or negative charge sites. The relevant reactions can be written as follows [26,27].

(1) $${\mathrm{POH}+H}^{+}\Leftrightarrow {\mathrm{POH}}_{2}^{+}$$

(2) $$\mathrm{POH}\Leftrightarrow {\mathrm{PO}}^{-}{+H}^{+}$$

where POH is the hydroxyl groups, P represents a surface site (in the present work, the represents Ga, Al or In, as shown in Figure 1). The hydroxyl groups (POH) are formed with P sites at the native oxide surface when the sensitive area contacts the aqueous solutions. The density of the POH groups can be modulated by chemically absorbing H^+^ and OH^−^ ions; thus, the surface charge type and density depend on the type and the concentration of the ions in solution, which is a function of the solution pH value [11]. When the concentration of H^+^ ions is higher than that of OH^−^ ions, the solution presents acidity. The POH groups tend to accept a proton and become protonated hydroxyls (POH^+^) acting as acceptors and resulting in positive charges at the insulator surface [28], represented by Equation (1). The 2DEG density will increase due to the surface positive charges’ gate effect, and hence, the current of the device will increase. On the other hand, in the aqueous alkaline solution, the concentration of OH^−^ ions is higher than that of H^+^. The reaction in the equilibrium Equation (2) becomes dominant; most of the POH groups release a proton and become PO^−^, as donors. The negative charges are formed at the surfaces, under the gate effect, reducing the 2DEG density and increasing the resistivity.

The density of 2DEG of GaN-based HETMs can be calculated by the following equation [25]:

(3) $$\begin{array}{l} n_{s}(x)=\sigma_{ABN/GaN}(x)/e-(\varepsilon_{0}E_{F}/e^{2})(\varepsilon_{ABN}(x)/d_{ABN}+\varepsilon_{GaN}/d_{GaN})-\\ \left(\varepsilon_{0}\varepsilon_{ABN}(x)/d_{ABN}e^{2})(e\phi_{ABN}(x)+E_{F}(x)-\Delta E_{ABN}^{c}(x)\right) \end{array}$$

where *ε*_0_ is the vacuum dielectric constant, *ε_GaN_* and *ε_ABN_* (*x*) are the relative dielectric constants (the ABN denotes AlGaN, InGaN or AlInN) and *d_ABN_* and *d_GaN_* are the thicknesses of the barrier layer and the buffer layer, respectively. *E_F_* is the position of the Femi level with respect to the GaN conduction-band-edge close to the GaN/substrate interface $e\emptyset_{ABN}\left(x\right)$ is the Schottky barrier of the gate contacting on top of the barrier, which would have no contribution to our devices because they are gateless. $E_{ABN}^{c}$ is the conduction band, and ∆$E_{\mathrm{ABN}}^{c}$ is the conduction band discontinuity between AlGaN and GaN. Obviously, the density is distinctly dependent on *E_F_*, which is adjusted by the surface charge states associated with the band bending modulation.

Specific to our pH sensing based on the current, the gateless AlInN/GaN or AlGaN/GaN device is equivalent to a resistor. Considering the effect of the interface charge between the protection layer SiN layer and AlGaN surface and the modulation of 2DEG density by the solution can be deemed as an equivalent gate effect generated by the gate voltage, *I_ds_* can be expressed as follows at the linear working region of *I_ds_* vs. *V_ds_* for our devices, as shown in Figure 2:

(4) $$I_{ds}=\frac{\varepsilon \mu W}{dL}(V_{g}-V_{off})V_{ds}$$

(5) $$R=L/(eun_{s}W)$$

with *ε* the dielectric constant of the barrier layer and μ the mobility of the electron. *W* is the open gate width; *L* is the open gate length; *V_g_* is equivalent gate voltage; *V_off_* is threshold voltage; *V_ds_* is drain voltage; *d* is the thickness of the barrier layer; *n_s_* is the density of the 2DEG in the channel. Alternately, Equation (4) means a linear correlation between *I_ds_* and *V_g_*, so dependent on the pH value of the solution. Besides, it is demonstrated that the *I_ds_* and therefore the slop of *I_ds_* with respect to the *V_ds_* is proportional to the ratio of *ε*/*d*, which naturally explains why the AlInN/GaN device has better performance in comparison with the AlGaN/GaN device, due to the higher *ε* and smaller *d* in the AlInN case.

According to Formula (4), increasing the ratio of open the gate width and length (*W*/*L*) can improve the drain current *I_ds_*, thus increasing the variation of *I_ds_* at different pH values, suggesting higher sensitivity. However, in our measurements, too large *W* or too short *L* does not mean high sensor sensitivity, as concluded from Figure 2. Normally, the current variation of *I_ds_* with different pH value solutions ((∆*I_ds_*/∆pH) not only depends on the device size, but also the geometry of the sensing area. In addition, based on Formulas (4) and (5), we can obtain the sensing area potential change and 2DEG density change. For example, when the solution pH value changes from 4–6, we can obtain Δ*R* (the change of the channel resistance) from Formula (5). The change of the 2DEG density (Δ*n_s_*) can be obtained also from the channel resistance formula, and the potential change due to the surface charge can be calculated, as well. For the AlGaN/GaN device, the change of potential Δ*ψ* is equal to 2.31 mV and Δ*n_s_* is equal to 6.46 × 10^9^ cm^−2^; for the AlInN/GaN device, Δ*ψ* is equal to 7.89 mV and Δ*n_s_* is equal to 2.21 × 10^10^ cm^−2^. From the results of Δ*ψ* and Δ*n_s_*, it can be known that AlInN/GaN device has higher variation. That is similar to the earlier discussion. From these measurement results and analysis, increasing the sensing area and *W*/*L* were not necessarily enhancing the device sensitivity due to the channel resistance decrease.

## 5. Conclusions

Comparative investigations on the pH value sensing performances of two GaN-based heterostructure devices, i.e., AlInN/GaN and AlGaN/GaN, were carried out in the present work. Both of the two kinds of devices demonstrate obvious current response to the loading of acidic and alkaline solutions at the linear working region of I–V. Good linear dependence of the current variation on the pH value was realized in both of the two kinds of devices. Notably, the AlInN/GaN devices exhibited much higher pH value sensitivity, compared with the AlGaN/GaN devices. Furthermore, experiments indicated that both of the two kinds of devices have a fast response speed and good stability of pH sensing, as well as good chemical resistance to the attacking solutions, and again, the AlInN/GaN devices displayed better performances. These results distinctly suggest that devices, especially the AlInN/GaN heterostructure devices, are applicable as practical pH value sensors.
